# Unsociability and social adjustment in Chinese preschool migrant children: the moderating role of receptive vocabulary

**DOI:** 10.3389/fpsyg.2024.1259975

**Published:** 2024-05-15

**Authors:** Jingjing Zhu, Shuhui Xiang, Xiaoqi Yin, Yan Li

**Affiliations:** Shanghai Institute of Early Childhood Education, Shanghai Normal University, Shanghai, China

**Keywords:** unsociability, receptive vocabulary, social adjustment, preschool migrant children, China

## Abstract

Peer relationships play an indispensable role in the social, emotional, and cognitive development of children. However, children exhibiting social withdrawal, such as unsociability, may face challenges in social adjustment. In collectivistic cultures like China, unsociability may be perceived negatively, aligning poorly with collective norms. The objective of the present investigation was to examine the associations between unsociability, receptive vocabulary, and indicators of social adjustment in a cohort of young migrant children residing in urban regions of mainland China. The study mainly aimed to investigate the potential moderating influence of receptive language on these associations. The study involved 148 young children (82 boys, 66 girls, *M_age_* = 62.32 months, *SD* = 6.76) enrolled in preschools or kindergartens in Shanghai, People’s Republic of China. Multiple sources of assessment were utilized, encompassing evaluations from mothers (about child unsociability), teacher (assessing social adjustment), and standardized tests (measuring receptive vocabulary). The results indicated that the relations between unsociability and peer exclusion were more positive among children with lower levels of receptive vocabulary but not significant for children with higher levels of receptive vocabulary. Similarly, the relations between unsociability and peer exclusion were more negative among children with lower levels of receptive vocabulary but not significant for children with higher levels of receptive vocabulary. Thus, this study informs us about how receptive vocabulary is jointly associated with unsociable children’s development. As well, the findings highlight the importance of considering the meaning and implication of unsociability in Chinese culture.

## 1 Introduction

Social adjustment refers to how an individual aligns their physiological and psychological well-being with the societal expectations for achieving developmental milestones, serving as a tangible manifestation of preschool children’s socialization outcomes (Cavell, 1990; Wang et al., 2023). Peers are essential and unique players in children’s social, emotional, and cognitive development (Rubin et al., 2015). Rubin et al. (2009) stated that children who are frequently removed from peer groups, also known as social withdrawal, have a heightened risk for socioemotional difficulties and may miss out on opportunities for developing social skills and building relationships. Asendorpf (1990) has identified shyness and unsociability as social withdrawal subtypes. Shy children are believed to face an approach-avoidance motivation conflict from a motivational standpoint, wherein their feelings of social unease inhibit their desire for social connection (Asendorpf, 1990; Coplan et al., 2004). Different from shyness, unsociability is often characterized as expressing lower social approach motivation and lower social avoidance motivation (Asendorpf, 1990). Specifically, it pertains to a child’s absence of inclination toward social activities, without actively opposing the involvement of others (Coplan and Armer, 2007). Shyness (or shyness sensitivity) is frequently linked to social fear and anxiety (Coplan et al., 2004). It is commonly defined as the desire to engage with peers tempered by the reluctance to participate due to anxiety or fear (Coplan et al., 2018). Conversely, unsociable children are characterized by a fearless preference toward solitary activities, often described as having an affinity for solitary behavior (Coplan et al., 2018). Consequently, shyness exhibits a more pronounced association with internalized emotions or sensitivities when compared to unsociability (Zhang and Eggum-Wilkens, 2018). Although shyness and unsociability have distinct constructs, studies showed that both are related to withdrawal behaviors and have negative correlations with social initiation and psychological engagement during interactions (Coplan et al., 2018). Consequently, it has become a prevalent approach to account for any shared variance in order to investigate their separate effects and consequences (Zhu et al., 2023).

Unsociable children often spend more time alone because they prefer to be alone (Asendorpf, 1990). In Western individualistic cultural contexts, unsociability is considered developmentally appropriate and is often described as a relatively benign form of social withdrawal (Coplan et al., 2018, 2019). For example, some studies conducted in Western contexts show that it is not associated with social adjustment in young children (Coplan et al., 2018, 2019). However, China is a collectivistic cultural society that highly emphasizes social interdependence and group affiliation (Chen and French, 2008). Children who exhibit unsociable behavior may be perceived as exhibiting characteristics that are counter to collective norms and self-centered, potentially leading to unfavorable responses from their peers and heightened vulnerability to adverse social outcomes (Chen et al., 2011). For example, some studies conducted among Chinese adolescents and preschool children found that unsociability was linked to internalizing problems and peer problems (Liu et al., 2017; Zhu et al., 2021, 2023). Linguistic competence is critical for interpersonal interactions, particularly language comprehension (i.e., receptive language) (Spitzberg and Cupach, 2011; White, 2014). Linguistic competence may therefore be a protective factor for the unsociable children’s development of social adjustment. Therefore, linguistic competence will also be explored in depth as a moderating variable in this study.

Migrant children are individuals under 18 whose current residence differs from the township or village specified in their hukou registration and who have been away from the place where they have been registered in the hukou for more than half a year (Han et al., 2023). The migrant children are a large group that cannot be ignored in China. Rural children in China are moving to cities with their families due to economic-driven migration, resulting in an increase in migrant children (Chen et al., 2009). Studies have shown that migrant children have lower levels of social adjustment and more social withdrawal behavior than non-migrant children (Rubin et al., 2014; Li et al., 2015). According to the “Annual Report on China’s Education for Migrant Children (2021–2022)” (2023), there are now 15.44 million preschool-aged migrant children in China, constituting 21.71% of all migrant children. Compared with school-age migrant children, preschool migrant children encounter great challenges, such as the transition of the parenting setting from home to kindergarten and the adjustment to an increasingly complex external environment (Huang and Wang, 2022). Thus, preschool migrant children may exhibit more unsociable behaviors. Therefore, in order to promote educational equity, this study targeted migrant children of preschool age.

Acknowledging that not all unsociable children would encounter social difficulties at school is imperative. A study on shy Chinese children found that receptive vocabulary buffers the adverse effects of shyness on children’s socioemotional functioning (Zhu et al., 2019). Thus, linguistic competence has the potential to serve as a protective element for unsociable children, thereby mitigating the likelihood of adverse outcomes. Therefore, this study investigates the potential protective role of receptive vocabulary to unsociability and social adjustment among Chinese migrant children. In addition, previous researches on unsociability and social adjustment were mainly focused on school-age children, with relatively little research on preschoolers, particularly Chinese migrant preschoolers. On the other hand, considering the role of culture in exploring unsociability and the importance of promoting equity in education, it is necessary to take into account the role of culture and the use of migrant children as research participants. Therefore, the primary objective of this research is to investigate the potential moderating influence of receptive vocabulary on the association between unsociability and social adjustment among Chinese preschool migrant children.

### 1.1 Overview of childhood unsociability in China

China is a unique collectivist society influenced by Confucianism, which is relational-centered rather than individual-centered (Wang and Liu, 2010). In such a society, the ultimate goal of people is to pursue harmony, which requires individuals to know their roles and act according to their positions. According to this belief, individuals who violate harmony are considered to be “morally lacking” (Bedford and Hwang, 2003). Although unsociable children do not avoid interaction with others, they have low social motivation. They prefer to be alone and do not initiate interaction (Coplan and Armer, 2007). In the Chinese context, children with unsociability were more likely to be labeled with negative words such as “anti-collective” and “selfish” (Chen et al., 2011). In a study conducted in China, it was found that peers were more likely to believe that unsociable children deliberately avoided participating in social interactions and would negatively impact on the classroom, as compared with socially competent and shy children (Ding et al., 2015a,b).

These negative evaluations may make unsociable Chinese children face more risk of negative adjustment than unsociable Western children (Liu et al., 2015). This negative effect is not only found in older children but also preschool children. Several research studies on Chinese preschool children have indicated a significant positive association between unsociability and peer exclusion, asocial behavior, and internalizing problems (Zhu et al., 2021, 2023). In addition, previous research on migrant children also found a positive correlation between unsociability and peer exclusion, internalizing problems, and loneliness, and a negative correlation between unsociability and prosocial behavior, interpersonal skills, peer preference, leadership, and academic achievement (Ding et al., 2020; Zhu et al., 2023).

### 1.2 Unsociability and language

Terms like “quiet,” “solitary,” or “reticent” are often included in the definitions of unsociability and its related structures (Eggum-Wilkens et al., 2020; Metin Aslan and Boz, 2022). Various theories have been proposed to explain the relationship between social withdrawal and language proficiency (Brinton and Fujiki, 1993; Beitchman et al., 1996; Goodyer, 2000). For example, previous researches suggested that children who display social problems may struggle to practice and develop language skills due to the limited opportunities of peer interactions (Brinton and Fujiki, 1993). Additionally, it has been proposed that socioemotional disorders (including withdrawal behavior) and language impairment may be linked due to the shared neurological foundations (Beitchman et al., 1996; Goodyer, 2000). Furthermore, some researchers have examined the relationship between unsociability and language (Ash et al., 2014; Fujiki et al., 2019). For example, Fujiki et al. (2019) founded that children with developmental language disorders tended to display more withdrawn behavior due to unsociability than typically developing children. In addition, Ash et al. (2014) revealed that English language learner children (no-native English speaking) in an English environment also do not have a significant increase in unsociability, compared native English speaking children. It means the adverse language environment may have less influence on increasing or decreasing children’s unsociability (Ash et al., 2014).

However, this current researches on the relationship between unsociability and language mainly focuses on the influence of language disorders or adverse language environments on children’s unsociable behavior. In addition, these studies are mainly carried out or proposed in the context of Western culture, but there are few studies on the correlation between unsociability and language in Chinese cultures. Meanwhile, some studies have shown that unsociable children show different social adjustments both in Chinese and Western cultures (Liu et al., 2015). For example, Ding et al. (2015a,b) have shown that Chinese preschool children preferred to play with peers who have high levels of social competence, followed by shy children and, finally, unsociable children. It indicates that unsociable children may have some difficulties in forming relationships with their peers. Therefore, there is a clear need to elucidate further and understand the link between unsociability and language in Chinese cultures.

### 1.3 Language as a protective factor

It is not necessarily the case that all unsociable children would encounter challenges regarding their social adjustment. Researchers have directed their attention toward comprehending the potential variables that could intensify or alleviate the probability of negative adjustment in unsociable children, including parental and child factors such as attachment, parenting behavior, behavioral inhibition, and resilience (Ding et al., 2015a,b; Chen and Santo, 2016; Zhu et al., 2021, 2023). Previous research conducted on Chinese preschool migrant children also found that children’s resilience significantly moderates the association between unsociability and social adjustment, and resilience served as a protective factor for the social adjustment of unsociable preschool migrant children in China (Zhu et al., 2023). Therefore, this study aims to investigate the social adjustment of preschool migrant children in China who are unsociable as well as any potential moderating factors in the links between unsociability and social adjustment.

Several researchers have explored the connection between social withdrawal and linguistic competence. Coplan and Evans (2009) have elucidated various theoretical mechanisms that may underpin the relationship between these two constructs. One possible mechanism is that shy children tend to speak less due to concerns about social evaluation, resulting in fewer opportunities to practice and enhance their language skills (e.g., Evans, 1993, 1996), potentially leading to diminished linguistic competence. Another perspective suggests that children with poorer language skills might encounter more difficulties in peer interactions, stemming from their weaker socio-communicative skills, which could contribute to increased shyness (Smith Watts et al., 2014). Blankson et al. (2011) also emphasize that linguistic competence comes from the child’s interaction with the environment. In other words, to some extent, social withdrawal impedes a person’s ability to learn new things. Consequently, a negative correlation may exist between social withdrawal and linguistic competence.

Peer experiences are greatly influenced by their social communication skills (Kaczmarek, 2002), and studies have indicated that children with language disorders have higher levels of unsociability than typically developing children (Fujiki et al., 2019). Therefore, we have reason to infer that language skills may buffer negative social adjustment in unsociable children. This assertion stems from the fact that difficulties in social interaction are the leading cause of difficulties in social adjustment in unsociable children (Rubin et al., 2009).

The current research on the protective effect of linguistic competence on the social adjustment of children with social withdrawal mainly focuses on shy children. For example, the association between shyness and socioemotional adjustment can be moderated by expressive vocabulary, according to a study on Canadian preschool children (Coplan and Armer, 2005). Expressive vocabulary may be boosting shy children’s confidence in peer interaction. Another study of shy preschool children in China found that higher levels of receptive vocabulary can mitigate the negative effects of shyness on children’s socioemotional functioning (Zhu et al., 2019). However, preschool children with lower levels of linguistic competence will have stronger correlation between shyness and adjustment difficulties (Zhu et al., 2019).

Supporting linguistic competence is indispensable to consolidating social skills and relationships within groups (Hay et al., 2004). Receptive vocabulary refers to the most fundamental meaning that children can understand. It is the cornerstone of children’s language building and plays a crucial role in communicating with others (Scarborough, 2001). Studies have shown that linguistic competence had the protective effect of on the social adjustment development of shy children (Zhu et al., 2019). Furthermore, since shyness is closely related to unsociability (Coplan et al., 2018), it is inferred that linguistic competence can also protect the social adjustment of unsociable preschool children. It is worth noting that linguistic competence is highly valued in traditional Chinese society (Wang and Liu, 2010). Thus, language skills may be essential for the social adjustment results of unsociable children in Chinese culture. However, there have been limited studies exploring the protective impact of language skills on the development of social adjustment in unsociable children, especially in Chinese culture. Therefore, exploring the protective effect of receptive vocabulary on the social adjustment results of children with unsociability in the Chinese context is necessary.

#### 1.3.1 The present study

The main goal of the current study was to investigate any potential moderating roles for receptive vocabulary in the link between unsociability and social adjustment in preschool migrant children in China. The selection of receptive vocabulary as a language component is based on its more robust association with linguistic competence than language performance. In order to attain the objective, the study evaluated the variables of unsociability, receptive vocabulary, and social adjustment indicators (e.g., peer exclusion, internalizing problems) in a substantial cohort of young children in China using multisource assessments (i.e., mother-rated, standardized tests, teacher-rated). We sought to reduce the effect of unfamiliar testing environments on the performance of unsociable children on language tests by evaluating receptive vocabulary in children’s classrooms (Crozier, 2001).

Furthermore, drawing from the existing literature, it is anticipated that there will be a positive correlation between unsociability and both peer exclusion and internalizing problems (Ding et al., 2020; Zhu et al., 2023). In contrast, a negative correlation is expected between unsociability, prosocial behavior, and social skills (Zhu et al., 2023). Given that few empirical studies have addressed the role of unsociability on language skills, we have yet to formulate a specific hypothesis regarding the negative correlation between unsociability and the ability to comprehend vocabulary. Our hypothesis posits that the receptive vocabulary level would influence the correlation between unsociability and adjustment difficulties. The present study found that the association between unsociability and the maladjustment index was weakened among children exhibiting elevated levels of receptive vocabulary. Conversely, the relationship was intensified among children displaying lower levels of receptive vocabulary.

## 2 Materials and methods

### 2.1 Participants

The study involved a sample of 148 preschoolers (*M_age_* = 62.32 months, *SD* = 6.76) who were migrants, comprising 82 boys and 66 girls. Shanghai is among the cities in China with the highest population of migrant children, and they predominantly enroll in public schools (Zhu and Ye, 2022). Therefore, to effectively obtain samples of migrant children, we collected samples of children from two public kindergartens in Shanghai and screened samples of migrant children by the children’s hukou location. Furthermore, it is noteworthy that the children who participated in this research belong to the Han ethnic group, which constitutes over 92% of the total population of China. The data indicate that a proportion of approximately 21.6% of the mothers and 24.3% of the fathers have attained a high school education. Furthermore, approximately 39.9% of the mothers and 27% of the fathers have completed tertiary education. In addition, approximately 35.1% of the mothers and 41.2% of the fathers have received a bachelor’s degree. In comparison, a smaller proportion of approximately 3.4% of the mothers and 7.4% of the fathers have attained a postgraduate degree.

### 2.2 Measures

#### 2.2.1 Maternal ratings

The Chinese version of the Child Social Preference Scale (CSPS; Coplan et al., 2004; Li et al., 2016) was completed by the mothers of the children. This investigation centered on the subscales utilized to evaluate unsociability and shyness. The unsociability scale includes 4 items (e.g., “My child is just as happy to play quietly by his/herself than to play with a group of children”; Cronbach’s α = 0.65). And shyness scale includes 7 items (e.g., “child seems to want to play with others but is sometimes nervous to”; Cronbach’s α = 0.86). Mothers were asked to rate each item on a five-point scale (from 1 = “not at all” to 5 = “a lot”). Higher aggregated scores indicate greater levels of unsociability/shyness.

#### 2.2.2 Child assessments

The assessment of children’s receptive vocabulary was conducted through the Peabody Pictures Vocabulary Test (PPVT-III) in its Chinese version (Dunn and Dunn, 1997). The Peabody Picture Vocabulary Test-III comprises a total of 204 items, with each item featuring a set of four pictures. Each item was visually presented on a quadrant, prompting the child to discern the most appropriate picture corresponding to the orally presented word. The experimental procedure involved groups of eight. Testing was terminated if a child committed over six errors in a block of eight items. The ultimate scores for receptive vocabulary ranged from 0 to 204 and were determined by deducting the total number of incorrect and omitted responses from the overall number of items. A higher score indicated better receptive-language abilities, as demonstrated in previous studies (Blankson et al., 2011). Empirical evidence suggests that the reliability and validity of the PPVT-III have been demonstrated in Chinese children (Cheung and Elliott, 2017; Zhu et al., 2021).

#### 2.2.3 Teacher ratings

The teachers were requested to fulfill the Chinese version of the Child Behavior Scale (CBS; Ladd and Profilet, 1996; Zhu et al., 2018), which comprises 35 items rated on a three-point scale (ranging from 1 = “does not apply” to 3 = “certainly applies”). This study placed particular emphasis on the subscales that evaluated prosocial behavior (consisting of 6 items, such as “prefers to play alone; keeps peers at distance,” with a Cronbach’s α = 0.88) and peer exclusion (comprising of 7 items, such as “ridiculed by peers,” with a Cronbach’s α = 0.93). Zhu et al. (2018) has demonstrated the reliability and efficacy of the CBS in the context of young Chinese children.

The Social Skills Rating System (SSRS; Gresham and Elliot, 1990; Zhu et al., 2017) was administered to teachers. The SSRS comprises 40 items rated on a three-point scale ranging from “never” to “always.” The study emphasized the subscales that evaluated interpersonal skills (consisting of 11 items, such as “Ease of forming friendships,” with a Cronbach’s α = 0.92) and internalizing problems (comprising of 4 items, such as “Displays signs of loneliness,” with a Cronbach’s α = 0.66). The reliability and efficacy of the SSTRS have been demonstrated in young Chinese children (Zhu et al., 2017).

### 2.3 Analytical strategy

The statistical software package SPSS 26 was utilized for conducting data analysis. The initial statistical procedures comprised a set of *t*-tests to investigate disparities between genders and associations among the variables under examination. Subsequently, the moderating effects were analyzed using Hayes’ (2022) PROCESS macro, employing nonparametric bootstrapping with 1,000 resamples. This study utilized a 95% bias-corrected confidence interval (CI) to investigate the notable effects (Preacher and Hayes, 2008). The Johnson-Neyman (J-N) technique, as recommended by previous researchers (Bullock et al., 2018), was employed to investigate significant interactions. This technique was first introduced by Johnson and Fay (1950).

## 3 Results

### 3.1 Preliminary analyses

Table 1 displays the descriptive statistics and intercorrelations of all variables examined in the study. The t-tests yielded statistically significant findings, revealing notable gender disparities in prosocial behaviors (*M_boy_* = 2.25, *SD* = 0.57; *M_girl_* = 2.45, *SD* = 0.52, *t* = −2.27, *p* = 0.03), peer exclusion (*M_boy_* = 1.23, *SD* = 0.43; *M_girl_* = 1.07, *SD* = 0.20, *t* = 2.92, *p* = 0.004), and interpersonal skills (*M_boy_* = 1.36, *SD* = 0.44; *M_girl_* = 1.57, *SD* = 0.36, *t* = −3.11, *p* = 0.002). Children’s age was also significantly and positively correlated with prosocial behavior, interpersonal skills, and receptive vocabulary. There was a significant and positive correlation between maternal education and the child’s receptive vocabulary. Subsequent analyses involved controlling for gender, age, and maternal education.

**Table 1:** Descriptive statistics and intercorrelations for all study variables.

Variables	1	2	3	4	5	6	7	8	9	10
Gender	–	−0.06	−0.03	0.01	−0.08	0.10	0.19^*^	−0.22^**^	0.25^***^	−0.14
Age		–	0.03	−0.07	−0.10	0.52^***^	0.46^***^	0.01	0.19^*^	0.10
Parental education			–	0.02	0.07	0.11	0.10	0.04	0.08	0.09
Shyness				–	0.63^***^	−0.19^*^	−0.27^***^	0.20^*^	−0.30^***^	0.24^***^
Unsociability					–	−0.29^***^	−0.29^***^	0.25^***^	−0.32^***^	0.15^+^
Receptive vocabulary						–	0.53^***^	−0.22^**^	0.50^***^	−0.19^*^
Prosocial behavior							–	−0.51^***^	0.63^***^	−0.25^***^
Peer exclusion								–	−0.65^***^	0.35^***^
Interpersonal skills									–	−0.42^***^
Internalizing problems										-
*M*		62.32	2.32	2.26	1.72	73.02	2.34	1.16	1.45	0.19
*SD*		6.76	0.93	0.76	0.57	28.98	0.56	0.36	0.42	0.30

^***^p < 0.001; ^**^p < 0.01; ^*^p < 0.05; ^+^*p* < 0.10.

As indicated in Table 1, parent-rated unsociability was significantly and negatively associated with teacher-rated prosocial behavior, interpersonal skills, and self-reported receptive vocabulary and was significantly and positively associated with teacher-rated peer exclusion but was not significantly associated with internalizing problems. In contrast, receptive vocabulary was significantly and positively with prosocial behavior and interpersonal skills and was significantly and negatively associated with peer exclusion and internalizing problems.

### 3.2 Unsociability, receptive vocabulary, and social adjustment

The principal objective of the present investigation was to scrutinize the moderating influence of children’s receptive vocabulary on the associations between unsociability and indicators of social adjustment. In order to achieve the objective, this study examined the effects of unsociability and vocabulary, as well as their interaction, on the outcome variables (i.e., prosocial behavior, peer exclusion, interpersonal skills, internalizing problems). The study also controlled for child gender, child age, and parental education. The SPSS PROCESS macro was utilized for conducting the analyses (Hayes, 2012).

Results are displayed in Table 2. With the additional control variables, these findings differed significantly from the correlation analyses. For example, unsociability was no longer significant with each outcome variables. Moreover, the significance of receptive vocabulary was strengthened with each outcome variable except peer exclusion. Of particular interest, significant interaction effects between unsociability and receptive vocabulary were found concerning peer exclusion and significant borderline effects between unsociability and receptive vocabulary were found concerning interpersonal skills (*p* = 0.05). And the interaction between unsociability and receptive vocabulary has no significant effect on prosocial behavior and internalizing problems.

**Table 2:** Effects of unsociability and receptive vocabulary (controlling for child gender, child age and parental education) in relation to indices of social adjustment.

Socioemotional variables				
Predictor	B	SE	*t-value*	95%CI
*Prosocial behavior*				
Unsociability	−0.11	0.09	−1.23	[−0.28, 0.06]
Receptive vocabulary	0.28	0.08	3.43^***^	[0.12, 0.44]
Unsociability × RV	0.07	0.07	1.08	[−0.06, 0.20]
*Peer exclusion*				
Unsociability	0.15	0.10	1.47	[−0.05, 0.35]
Receptive vocabulary	−0.19	0.10	−1.98^+^	[−0.38, 0.00]
Unsociability × RV	−0.19	0.08	−2.43^*^	[−0.34, −0.04]
*Interpersonal skills*				
Unsociability	−0.10	0.09	−1.16	[−0.28, 0.07]
Receptive vocabulary	0.43	0.08	5.12^***^	[0.26, 0.60]
Unsociability × RV	0.14	0.07	1.97^+^	[0, 0.27]
*Internalizing problems*				
Unsociability	−0.05	0.10	−0.49	[−0.25, 0.15]
Receptive vocabulary	−0.29	0.10	−2.97^***^	[−0.48, −0.10]
Unsociability × RV	−0.04	0.08	−0.56	[−0.20, 0.11]

RV, receptive vocabulary. ^***^p < 0.001; ^**^p < 0.01; ^*^p < 0.05.

Following the recommendations of Hayes and Matthes (2009), the Johnson-Neyman (J-N) method (Johnson and Fay, 1950) was employed to investigate the significant interactions, with all predictors being standardized for the analyses. The utilization of this methodology enabled us to estimate a “region of significance” of the simple slope of a predictor, which is contingent on the value of the continuous moderator.

**Figure 1:**
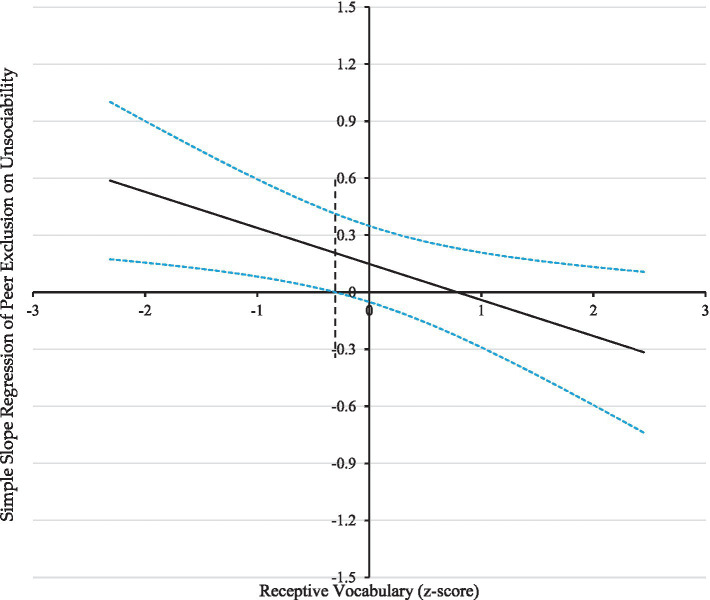
Johnson-Neyman regions of significance and confidence bands for mother-rated unsociability along receptive vocabulary in relation to peer exclusion. The dashed vertical line indicates the point along receptive vocabulary at which the unsociability regression coefficient transitions from statistical significance (left of dashed vertical line) to non-significance (right of dashed vertical line). The value of the dashed vertical line is −0.30. The solid diagonal line represents the regression coefficient for unsociability along receptive vocabulary. The dashed diagonal lines are confidence bands—upper and lower bounds of 95% confidence interval—for the unsociability regression coefficient along receptive vocabulary.

**Figure 2:**
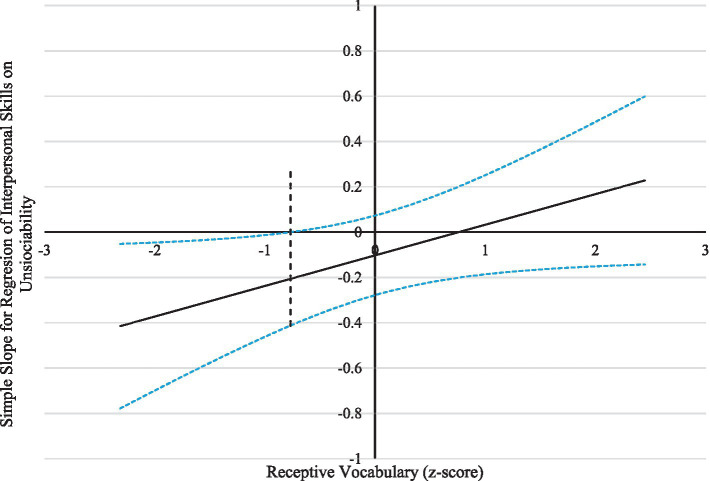
Johnson-Neyman regions of significance and confidence bands for mother-rated unsociability along receptive vocabulary in relation to interpersonal skills. The dashed vertical line indicates the point along receptive vocabulary at which the unsociability regression coefficient transitions from statistical significance (left of dashed vertical line) to nonsignificance (right of dashed vertical line). The value of the dashed vertical line is −0.77. The solid diagonal line represents the regression coefficient for unsociability along receptive vocabulary. The dashed diagonal lines are confidence bands—upper and lower bounds of 95% confidence interval—for the unsociability regression coefficient along receptive vocabulary.

The findings are visually depicted in Figures 1, 2. The observed outcomes exhibited a similar pattern for both variables under consideration. A significant and positive correlation was observed between unsociability and peer exclusion when the receptive vocabulary score was less than −0.30. The phenomenon above exhibited a diminishing trend with increased receptive vocabulary scores. It was observed that when the vocabulary scores were equal to or exceeded −0.30, the correlation between unsociability and peer exclusion was no longer significant. In a similar vein, it was observed that a significant and negative correlation existed between unsociability and interpersonal skills when the scores for receptive vocabulary were less than −0.77. Nevertheless, the related factors were mitigated so that they no longer achieved statistical significance as vocabulary scores attained and surpassed −0.77. These results suggest a protective mechanism for receptive vocabulary in the associations between unsociable behavior and indicators of social adjustments, such as peer exclusion and interpersonal skills.

It is worth mentioning that we also tested the moderating role of gender between unsociability and indicators of social adjustment, considering the correlation between gender and indicators of social adjustment (prosocial behavior, peer exclusion, and interpersonal skills). The results indicated there was no significant two-way interaction effect (gender × shyness) for all indicators of social adjustment.

## 4 Discussion

The objective of the current study investigation was to analyze the correlations among unsociability, receptive vocabulary, and indicators of social adjustment in a cohort of young migrant preschoolers in China. In addition, our study examined the potential moderating effects of receptive vocabulary on the correlations between unsociability and adjustment. The findings showed that unsociability was linked to all expected social adjustment outcomes. Furthermore, receptive vocabulary could buffer the associations between unsociable behavior and indicators of social adjustments, such as peer exclusion and interpersonal skills.

### 4.1 Implications of early childhood unsociability in China

The results of this study indicate that unsociability in Chinese preschool migrant children is significantly negatively correlated with prosocial behavior and interpersonal skills, significantly positively correlated with peer exclusion, and marginally positively correlated with internalizing problems. This result is consistent with previous Chinese studies on preschool children (Zhu et al., 2023), but differs from the findings from studies conducted in the West (Sette et al., 2017; Coplan et al., 2018). It may be related to Chinese culture emphasizing harmonious interpersonal relations and collectivism (Huang and Wang, 2022).

Notably, the results of this study differ from those of previous studies on Chinese preschoolers studying in cities (without restricted household registration) (e.g., Zhu et al., 2021). This study suggested that children’s unsociability and interpersonal skills have a significantly positive correlation, which is different from the results of some Chinese studies. For example, Xie et al. (2020) conducted a study on non-migrant preschool children in China and found that unsociability was non-significantly with children’s interpersonal skills. Unlike non-migrant children, migrant children must face psychological and social-culture adjustment problems (including lifestyle and interpersonal adjustment) (Kong et al., 2015). These findings of this research further support that compared with non-migrant unsociable preschoolers, migrant unsociable preschoolers have poor interpersonal skills and may suffer more social adjustment difficulties.

In addition, this study also found that children’s unsociability and internalizing problems have a marginal positive correlation (0.1 < *p* ≤ 0.05), which is different from the previous studies. For example, Zhu et al. (2021) conducted a study on Chinese preschool children and found that unsociability was significantly and positively correlated with children’s anxious-fearful behavior (*p* < 0.01). Unlike non-migrant children, Migrant children contact their hometowns (Liu and Fang, 2013) and can obtain identity from the urban group and the group where their hometowns are. On the one hand, unsociable children usually prefer to be alone and concentrate on their affairs, but they are not averse to getting along with others (Coplan and Armer, 2007). Adults in underdeveloped areas of China usually think that such children are “lovable and sensible.” On the other hand, people’s stereotype of “everything is good” in big cities (Zhang, 2011) may also make adults in children’s hometowns pay more attention to the advantages of migrant children to affirm the education in big cities, making them become “someone else’s children” (i.e., excellent children). Therefore, taking these two reasons together, unsociable migrant children may be able to acquire group identity from adults and children in their hometowns. Thereby they may have fewer internalizing problems than non-migrant children.

The results above contribute to the expanding corpus of research that indicates a correlation between unsociability and social adjustment in preschool-aged children residing in China. Moreover, it is becoming increasingly clear that Chinese children with unsociability may need our attention. Unsociable children are currently posing a risk of developing early peer relationship difficulties. The literature indicates that peer-related problems during the early stages of childhood pose a considerable risk for various adjustment challenges (Rubin et al., 2015). Liu et al. (2017) have presented evidence of a longitudinal association between negative peer experiences and the subsequent development of internalizing problems and learning difficulties in a cohort of urban primary school students in China. Additionally, early subclinical symptoms of internalizing problems have been linked to a heightened likelihood of subsequent socioemotional challenges (Feng et al., 2008). Despite being a less explored area, there is an increasing body of evidence indicating that subclinical anxiety symptoms during childhood are linked to comparable negative consequences in China, as reported by Liu et al. (2015) and Zhu et al. (2017).

### 4.2 Unsociability and receptive vocabulary

Unlike previous research results on shy and receptive vocabulary (Zhu et al., 2019), unsociability negatively correlates with preschool children’s receptive vocabulary. This finding is consistent with previous findings that unsociability positively correlates with language disorders, with children with language disorders often showing more unsociability (Fujiki et al., 2019).

Unlike the shy children, who showed higher social approach motivation, the unsociable children showed lower motivation (Coplan and Armer, 2007). Therefore, compared with shy children, unsociable children attach less importance to social interaction and are less inclined to exercise their communicative competence. In addition, children with unsociability may harm their interactions with other children if their initial levels of linguistic competence are low, so unsociable children are more likely to choose solitude and increase their unsociable behavior.

In addition, the participants of this study were migrant children, which may be one of the reasons why unsociability is significantly correlated with children’s receptive vocabulary. Studies have shown that migrant children have significantly lower levels of linguistic competence than non-migrant children (Wang and Zhuang, 2012; Liu et al., 2015). It may indicate that the migrant children themselves have a lower level of receptive vocabulary. However, the character of migrant children with unsociability ignores communication, reducing their chances to practice their linguistic competence and eventually decreasing their linguistic competence.

The results of this study provide more samples of the relationship between unsociability and linguistic competence of preschool children, especially children from non-Western cultures. In addition, the results of this study indicate that more opportunities should be provided for unsociable children to exercise their linguistic competence, and teachers and parents should consciously help them improve their linguistic competence.

### 4.3 Vocabulary as a protective factor for unsociable children

The principal objective of this investigation was to examine how receptive vocabulary functions as a moderator of unsociability and various adjustment indicators. The current investigation revealed a positive correlation between receptive language abilities, prosocial behavior, and interpersonal skills. Conversely, a negative correlation was observed between receptive language skills, peer exclusion, and internalizing problems. The findings presented in this study align with prior research indicating a correlation between children’s social adjustment functioning and their linguistic competence, as demonstrated by Kaczmarek (2002) and others.

This study’s findings indicate a significant relationship between unsociability and peer exclusion, which is moderated by receptive vocabulary. Notably, receptive vocabulary was marginally significant with peer problems in migrant children, which is slightly different from previous studies. For example, Zhu et al. (2019) found in their study of Chinese preschool children that receptive vocabulary can significantly predict the peer acceptance of preschool children. The reason for this difference may be that peers ostracize migrant children because of migration status (Zhang et al., 2020), so the effect of receptive words on their peer ostracism is insignificant. Additionally, a marginal relationship between unsociability and interpersonal skills is moderated by receptive vocabulary. Moreover, to put it differently, a correlation was observed between unsociability and peer exclusion and lower levels of interpersonal skills in individuals with limited receptive vocabulary. On the contrary, the connections above were weakened when considering elevated levels of receptive vocabulary.

The moderating roles of receptive vocabulary on the effects of unsociability on peer exclusion and interpersonal skills confirm the perspectives by Hay et al. (2004), that is, language is one of the basic skills to support the ability to get along with peers and the support of linguistic competence is indispensable to consolidate social skills and group relations. Vocabulary accumulation is the basis of children’s conversation with peers, and conversational competence is an element that influences children’s successful interaction with peers and acceptance by peers (Hay et al., 2004). In addition, a child’s ability to understand different words is essential to kindergarten preparation and can support socioemotional adjustment (Bierman et al., 2008). Therefore, when unsociable migrant children have higher levels of receptive vocabulary, they can have positive interactions with their peers because such positive feedback may lead to more interactions initiated by their peers.

Unsociable children pay less attention to social interaction and seldom initiate interactive behaviors (Coplan and Armer, 2007). Therefore, unlike shy children who receive positive feedback through receptive vocabulary, prosocial behaviors will increase, and internalizing problems will decrease (Zhu et al., 2019), receptive vocabulary has little impact on the prosocial behaviors and internalizing problems of children with unsociability. In addition, unsociable migrant children’s unsociable characters and connections with their hometowns may be able to get more attention and affirmation from adults and children in their hometowns. Therefore, the marginal correlation between unsociability and the internalizing problems of migrant children. It may also be one of the reasons why receptive vocabulary does not play a significant role in regulating the correlation between unsociability and internalizing problems.

To sum up, in order to help unsociable children to reduce the risk of social difficulties, teachers and parents should try their best to provide some chances to exercise children’s language skills, help children to improve their language skills, and then help children to consolidate their peer relationships better and establish a good interpersonal relationship.

### 4.4 Limitations, directions for future research, and implications

This study preliminarily supports the view that higher receptive-language skills are a protective factor in the social adjustment of migrant children with unsociability in China. Nonetheless, it is important to note that this research has several limitations. First, the inter-associations of this study prevent us from concluding the causal mechanisms underlying these relationships. It is imperative to take into account alternative explanations in this context. For example, teachers may exhibit a more favorable response toward unsociability with better linguistic competence and think they are better adjusted. Therefore, longitudinal studies are warranted further to elucidate the nature of these potentially complex associations. Second, there are significant regional variances in social and economic development, but this research only looked at preschool-aged migrant children in Shanghai, one of China’s most developed cities. Policies and structures for migrant children are relatively well-developed in Shanghai. Hence, the question of the generalizability of the study findings remains to be established. As a result, we can carry out studies in the future in other cities with various economic and cultural backgrounds. Furthermore, the current study exclusively relied on assessments conducted by mothers, teachers, and standardized tests, which may be affected by evaluator bias or errors. Therefore, future studies could consider multi-source evaluations (e.g., child self-report or peer assessment). Finally, it should be noted that young children’s language skills are not limited to receptive languages. Future research should continue to investigate the moderating role of other aspects of language (e.g., grammar, morphology) in non-Western cultures (e.g., China), as well as their relationship to unsociability and broader adaptive outcomes (e.g., academic achievement, teacher-children relationship).

Nonetheless, some implications for upgrading the educational practice levels can be drawn from these findings. In other words, while trying to improve the quantity and quality of unsociable children’s social interaction in school, our research results show that teachers should also pay attention to improving unsociable children’s language field. Previous studies have shown that children’s language skills can be improved through school interventions (Hulme et al., 2020). For example, the Nuffield Early Language Intervention (NELI; Bowyer-Crane et al., 2008) found that alternating daily group work and one-on-one sessions for children with weak oral linguistic competence can effectively improve children’s vocabulary and narrative skills.

In addition, given that parents are children’s first teachers, they also play an essential role in helping children improve their language skills. For example, Aram and Levin (2014) found that parents reading storybooks with preschool children three times a week significantly improved preschool children’s language skills (i.e., receptive and expressive vocabulary, listening comprehension, and definitions).

In conclusion, our findings complement previous studies (the older sample), suggesting that unsociability is associated with difficulties in social adjustment among migrant children in contemporary China, even in early childhood. In addition, the aforementioned findings offer the initial empirical support that receptive language skills protect young children from unsociability in this specific cultural milieu. Therefore, it will be essential to continue studying strategies and approaches suitable for Chinese culture to improve the early school experience of unsociable Chinese children.

## Data availability statement

The raw data supporting the conclusions of this article will be made available by the authors, without undue reservation.

## Ethics statement

The studies involving human participants were reviewed and approved by Shanghai Normal University. Written informed consent to participate in this study was provided by the participants legal guardian/next of kin.

## Author contributions

JZ: Conceptualization, Data curation, Investigation, Resources, Writing – review & editing. SX: Data curation, Investigation, Methodology, Writing – original draft, Writing – review & editing. XY: Writing – original draft. YL: Resources, Writing – review & editing.
